# Unbiased Rare Event Sampling in Spatial Stochastic Systems Biology Models Using a Weighted Ensemble of Trajectories

**DOI:** 10.1371/journal.pcbi.1004611

**Published:** 2016-02-04

**Authors:** Rory M. Donovan, Jose-Juan Tapia, Devin P. Sullivan, James R. Faeder, Robert F. Murphy, Markus Dittrich, Daniel M. Zuckerman

**Affiliations:** 1 Joint CMU-Pitt Ph.D. Program in Computational Biology, Pittsburgh, Pennsylvania, United States of America; 2 Department of Computational and Systems Biology, School of Medicine, University of Pittsburgh, Pittsburgh, Pennsylvania, United States of America; 3 Computational Biology Department, School of Computer Science, Carnegie Mellon University, Pittsburgh, Pennsylvania, United States of America; 4 Pittsburgh Supercomputing Center, Pittsburgh, Pennsylvania, United States of America; National Institutes of Health, UNITED STATES

## Abstract

The long-term goal of connecting scales in biological simulation can be facilitated by scale-agnostic methods. We demonstrate that the weighted ensemble (WE) strategy, initially developed for molecular simulations, applies effectively to spatially resolved cell-scale simulations. The WE approach runs an ensemble of parallel trajectories with assigned weights and uses a statistical resampling strategy of replicating and pruning trajectories to focus computational effort on difficult-to-sample regions. The method can also generate unbiased estimates of non-equilibrium and equilibrium observables, sometimes with significantly less aggregate computing time than would be possible using standard parallelization. Here, we use WE to orchestrate particle-based kinetic Monte Carlo simulations, which include spatial geometry (e.g., of organelles, plasma membrane) and biochemical interactions among mobile molecular species. We study a series of models exhibiting spatial, temporal and biochemical complexity and show that although WE has important limitations, it can achieve performance significantly exceeding standard parallel simulation—by orders of magnitude for some observables.

## Author Summary

Stochastic simulations (simulations where randomness plays a role) of even simple biological systems are often so computationally intensive that it is impossible, in practice, to simulate them exhaustively and gather good statistics about the likelihood of different outcomes. The difficulty is compounded for the observation of rare events in these simulations; unfortunately, rare events, such as state transitions and barrier crossings, are often those of particular interest. Using the weighted ensemble (WE) method, we are able to enhance the characterization of rare events in cell biology simulations, but in such a way that the statistics for these events remain unbiased. The histogram of outcomes that WE produces has the same shape as a naive one, but the resolution of events in the tails of the histogram is greatly improved. This improved resolution in rare event statistics can be used to infer unbiased estimates of long timescale dynamics from short simulations, and we show that using a weighted ensemble can result in a reduction in total simulation time needed to sample certain events of interest in spatial, stochastic models of biological systems.

This is a PLOS Computational Biology Methods paper.

## Introduction

Stochastic effects are of crucial importance in many biological processes, from protein dynamics [1], to gene expression [2], to phenotypic heterogeneity [3]. Unfortunately, due to the high computational cost of simulating complex stochastic biological systems, the effects of stochasticity on system response remain under-studied in realistic biological models.

From molecular to cellular scales, simulations of biological systems push the limits of our computational resources [4, 5]. Compromising between sampling power and model complexity will be a trade-off for the foreseeable future; for example, at atomistic resolution even the most powerful, specially designed supercomputers can simulate only modestly sized proteins at timescales that approach sufficiency for adequate sampling [6]. Similarly, models of cellular processes, though they omit entirely molecular-level details, are also constrained in complexity and realism by the need to perform adequate amounts of simulation in order to gather useful statistics [7]. Mixing scales in a simulation, though perhaps necessary for capturing the coupling across multi-scale networks, only makes this problem worse.

Enhanced sampling algorithms offer an attractive proposition: instead of compromising on model complexity in order to achieve well-sampled results, rather use simulation resources more effectively and extract more information given the same resources. Not surprisingly, there has been significant interest in sampling algorithms in the field of atomistic protein simulation, including umbrella and histogram sampling [8–10], path sampling methods, [11–17], and various flavors of replica exchange [18–21]. Arguably, such approaches have transformed the field of molecular simulation [6, 22, 23].

The essence of the present study is the extension of one successful enhanced sampling strategy for molecular simulation to spatially resolved cell-scale systems. Specifically, the weighted ensemble approach is a scale-agnostic method that is able to facilitate the enhanced sampling of a wide spectrum of stochastic simulations and non-Markovian processes [17], including Brownian dynamics [13], molecular dynamics [24], Monte-Carlo simulations of atomistic and coarse-grained protein dynamics [25, 26], chemical reaction networks [27], and as we demonstrate here, the spatially resolved stochastic reaction-diffusion processes used to simulate cellular processes. Weighted ensemble achieves its enhanced sampling by dividing up a model’s state-space into bins and maintaining an ensemble of trajectories with different weights that evenly sample these bins. This weighted ensemble is created by resampling the distribution of trajectories at fixed time intervals, spawning new simulations from trajectories that have wandered into unexplored regions and pruning them away if a region is overpopulated, in order to maintain even coverage of the space. This resampling process is exact, in the sense that it induces no bias in the estimates of equilibrium and non-equilibrium observables [17, 28]. Resampling at fixed time intervals lends the method some key benefits: it is trivially parallelizable, since trajectories run independently aside from interacting infrequently during resampling, and it is modular, needing no “under the hood” interaction with the underlying dynamics, rather requiring only intermittent reports of a progress coordinate.

Spatial heterogeneity can be crucial to accurately capturing the behavior of cell-scale biological systems, for instance in models of neuromuscular junction dynamics studied below [29]. Although simple models of biological signaling, where the molecules of interest are spatially homogeneous, or “well-mixed” are very common [30, 31], the assumption of spatial homogeneity may not always be justified; certain biological systems, while suitable for ignoring molecular structure, are not amenable to being modeled as spatially homogenous. Indeed, high resolution microscopy images of single cells show distinct patterns of localization for a wide variety of biomolecules [32–34], leading one to speculate if the well-mixed regime is the exception rather than the rule.

Here, we apply the weighted ensemble sampling procedure to decrease the cost of simulating spatial stochastic systems. After introducing our methodology, we present results for a toy diffusive binding system and two more complex systems: a cross-compartmental signal transduction model in a realistic cellular geometry and a model of an active zone in a frog neuromuscular junction. The flexibility and power of the WE method make it ideally suited for enhancing the sampling of these three diverse models.

## Methods

We employ the weighted ensemble sampling algorithm to manage multiple instances of particle-based kinetic Monte Carlo simulations of a given spatially resolved model of cellular signaling. We make use of a variety of software packages in our work, all of which are freely available via MMBioS.org.

### Weighted Ensemble

#### Basic weighted ensemble

The weighted ensemble sampling strategy achieves enhanced sampling by maintaining an ensemble of simulations running in parallel, distributed evenly across the configuration or state space of a system. To do this, the configuration space of the system is typically divided into different region, or “bins”, according to the values of some progress coordinate(s). The parallel ensemble of simulations is periodically paused, and each simulation is inspected to ascertain which bin it inhabits. Simulations in overpopulated bins are pruned away until a desired population is reached, and simulations in underpopulated bins are duplicated until a sufficient population is reached. After this brief resampling process, the ensemble of trajectories is restarted, and the native dynamics of the system continues, until it comes time to pause and resample again. By assigning each trajectory a statistical weight and conserving this weight during pruning and cloning operations, the ensemble remains unbiased, while efficiently sampling otherwise difficult to reach regions of configurations space [13].

**Fig 1:**
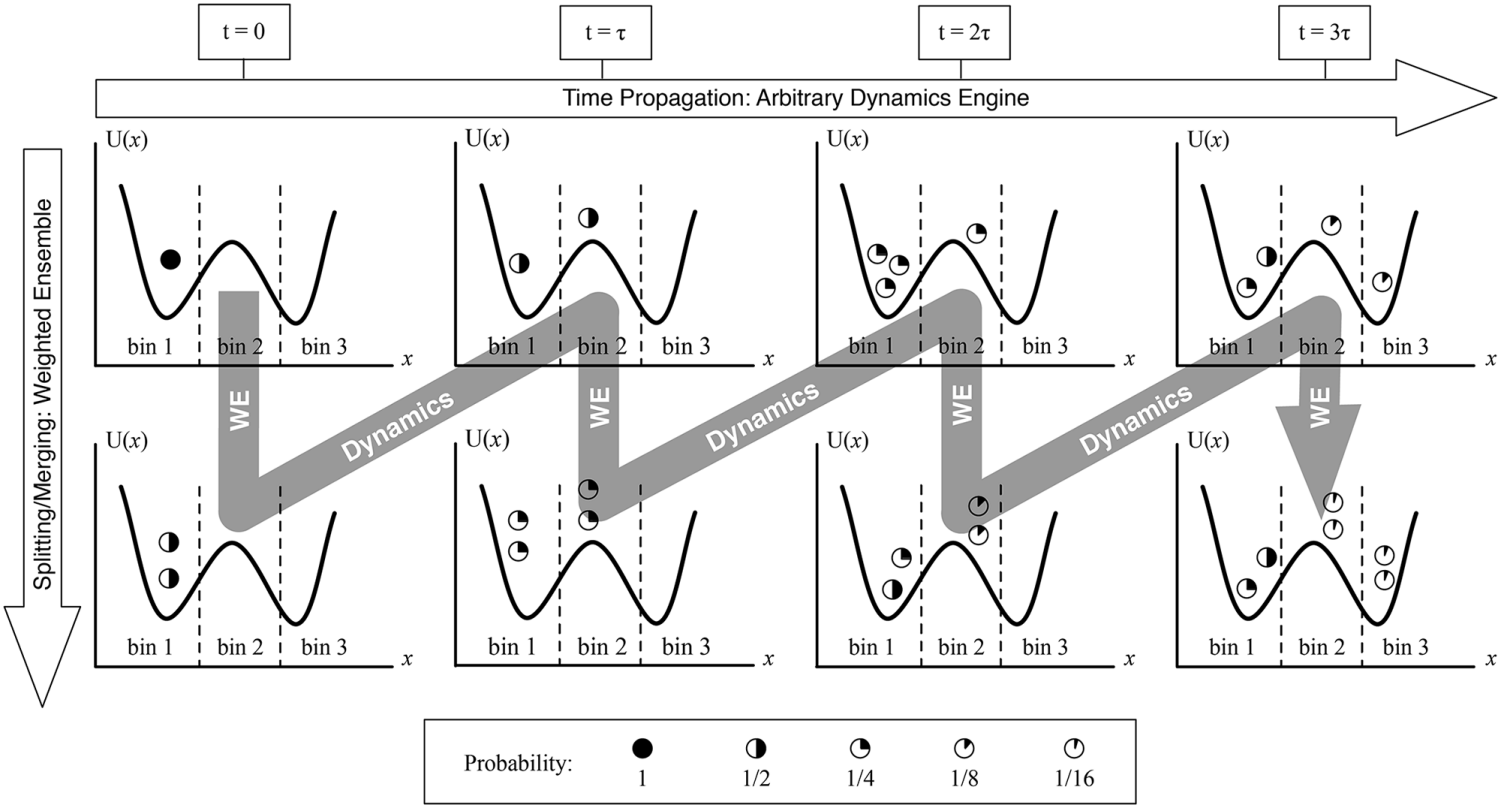
Weighted ensemble algorithm. One initial trajectory is iteratively resampled until its progeny trajectories are distributed equally around the state-space, according to user-defined bins. Excess trajectories are pruned if necessary. This process is statistically exact if the probabilistic weight of each trajectory is properly accounted for in the resampling (splitting/merging) process. In between splitting and merging events, each trajectory follows the natural dynamics of the system, and splitting events occur when a trajectory naturally wanders into an underpopulated region of space. Reprinted with permission from *Efficient stochastic simulation of chemical kinetics networks using a weighted ensemble of trajectories, The Journal of Chemical Physics 139: 115105*.

The essence of the weighted ensemble sampling procedure is encapsulated in Fig 1, where we have chosen to divide the example system along one coordinate into three bins, and have a target number of two trajectories in each bin. Before the simulation begins, the configuration space of the system must be considered, and typically a progress coordinate (or more than one) along which a trajectory can be tracked is selected. Although automated binning procedures have been developed [17, 35], we do not use them in the studies reported here. The configuration space of the system is divided into non-overlapping bins of the selected progress coordinate(s) that completely cover the configuration space. This division is usually done ahead of time, but “on the fly” modifications are also permitted [17]. In Fig 1, a one-dimensional projection of a system is shown, and the space is divided into three bins, which remain the same throughout the simulation. For efficient sampling, the progress coordinates chosen should be associated with a set of slowly varying and uncorrelated processes; additional progress coordinates tend to increase computing cost without a sufficient “payoff” in sampling. Additional slowly varying progress coordinates can speed up sampling of slow/rare processes in the system, but choosing progress coordinates that are uncorrelated is also important, because correlated coordinates are redundant in the variation of the system that they capture. The expense of maintaining bins full of trajectories increases drastically with the number of progress coordinates used, making it essential to use additional progress coordinates only when they are crucial to capturing new information about the system.

In the basic weighted ensemble procedure, a number of replicate trajectories are initiated from a chosen initial state, with weights summing to one, and are simulated for a short time *τ*. After that short time, the simulations are paused and inspected for progress along the chosen progress coordinates. If a trajectory has wandered into a new, previously unpopulated bin, that trajectory is replicated, and the statistical weight of that trajectory is divided among these “daughter” trajectories. If a bin becomes over-populated, trajectories are pruned and their weights are reassigned. After this pause in dynamics for resampling, the trajectories are restarted, and the entire process is iterated as desired.

The resampling strategy of WE is exact for arbitrary types of stochastic dynamics in any number of dimensions [17, 28]. Typically, when we divide the configuration space of the system into bins, we set a target number of trajectories for each bin; if, during one of the intermittent resampling events, the number of trajectories in that bin is greater or less than the target (but nonzero), we either up- or down-sample the trajectories in the bin to reach the target number, always accounting for the statistical weights of each trajectory. “Up-sampling” connotes spawning new trajectories, identical to the original but with the original trajectory’s statistical weight now split between the new and old trajectories. For instance, during the *t* = *τ* resampling event in Fig 1, the trajectory in bin 2, initially possessing a weight of 1/2, spawns an identical copy of itself, and the weight of the original trajectory is evenly divided so that the two resultant trajectories each have a weight of 1/4. “Down-sampling” is a pruning process, whereby trajectories are compared in a pairwise fashion and one is deleted based on a random process, with a likelihood of survival proportional to the statistical weight of the trajectory. For instance, during the *t* = 2*τ* resampling event in Fig 1, the three trajectories in bin 1 all have weight 1/4, so two of them are selected, and a random number draw (evenly weighted, since both trajectories have the same weight) decides which one remains. By these two simple processes, an ensemble of trajectories is created that evenly samples the state space of the system without bias [17].

The resampling process adds a small amount of computational overhead to the overall cost of sampling. This expense, however, is a small fraction of the total cost, provided that either the dynamics of the system are expensive to simulate, or the resampling interval is long compared to the timescale of the internal dynamics of the simulation, which we find is almost always the case in systems of interest. For instance, when using weighted ensemble to run simulations of molecular dynamics [24], large chemical kinetics networks [27], or the spatially resolved stochastic chemical kinetics studied here, the trajectories will typically run for a wall-clock time on the order of minutes or hours before being paused for resampling, while the resampling operation itself takes on the order of seconds. Indeed, the resampling arithmetic itself is trivial in complexity compared to the stochastic dynamics of the trajectories themselves, and most of the time spent during resampling is actually spent reading and writing to disk and starting and stopping trajectories (if the data are too large to store in memory). Like any enhanced sampling method, WE is worthwhile only for complex models exhibiting a wide variety of timescales.

**Fig 2:**
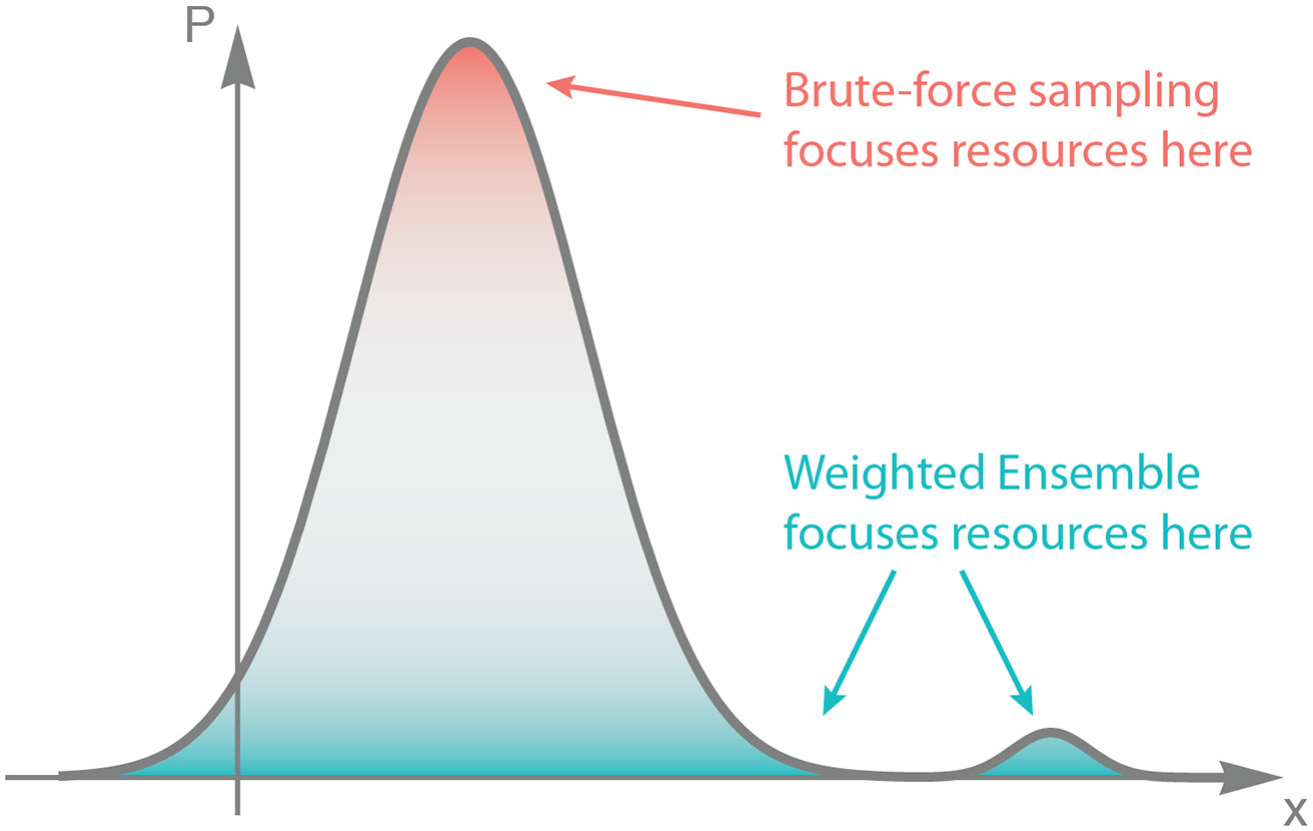
Distribution of sampling power. Brute-force sampling, by definition, concentrates sampling power on the most probable events. By contrast, weighted ensemble samples a distribution more evenly, and compared to brute-force it applies more resources to hard-to-sample regions of interest.

The benefit of this resampling process is that it facilitates the efficient, exact sampling of the system along the binned progress coordinates. As illustrated schematically in Fig 2, in a naive “brute-force” approach, where a number of independent trajectories are simulated and then compiled into a histograms of outcomes, the sampling power of the ensemble is concentrated about the peak of the distribution.

By definition, the peak contains the most probable events. Thus, certain parts of the configuration space are destined to be poorly sampled; if the true probability of a state being occupied is less than the inverse of the number of trajectories simulated, it is unlikely to be sampled even once. On the other hand, weighted ensemble decouples the number of trajectories in a region of configuration space from the probability of a trajectory to be there, and allows for a more even coverage of all regions of the underlying distribution. It should be noted that an even coverage of configuration space lends itself to efficient sampling only if the coordinate(s) along which this coverage is distributed are useful in characterizing the observables of interest [17]. That is, the payoff of using weighted ensemble sampling depends on one’s choice of progress coordinate and bins, and efficiently sampling certain regions of configuration space may prove unrewarding.

#### Calculation of mean first passage time with WE

The efficient sampling of low-probability regions of the (time-dependent) probability distribution of a stochastic system can be leveraged to extract unbiased estimates of long-timescale information about the system. Specifically, the Hill relation [36] provides a link between the mean first passage time (MFPT) between two states *A* and *B*, and the steady-state flux of probability (flow per unit time) between them:

$$\begin{array}{c} \mathrm{MFPT}\left(A\to B\right)=\frac{1}{\mathrm{Flux}(A\to B)}\;\;, \end{array}$$ (1)

where Flux(*A* → *B*) here refers to the probability (per unit time) that a trajectory, which at some point in the past originated in *A*, arrives at *B* for the first time. This relationship is exact (up to statistical noise) when the system exhibits a steady-state flow of probability from *A* to *B*. These two states *A* and *B* can be single micro-states, or large states composed of many smaller sub-states (e.g. weighted ensemble bins); they can also be arbitrarily defined independent of the WE bin boundaries.

A steady-state is achieved when the probability distribution of the system is constant in time. That is, for each sub-state *i* in the system, in a given time period the total flow of probability into *i* from the other sub-states is equal to the flow of probability out of *i* into other sub-states. In terms of the steady-state probabilities *p*_*i*_ of each sub-state, and the transition probabilities *k*_*ij*_ between sub-states (in an arbitrary, but fixed, time interval), the steady-state condition is given by

$$\begin{array}{c} \forall i\;:\;\sum_{j}k_{ji}p_{j}=\sum_{j}k_{ij}p_{i}\;. \end{array}$$ (2)

The conditional probabilities *k*_*ij*_ can be estimated from WE, and the *p*_*i*_ values can then be inferred by solving the linear system in Eq 2. The Flux(*A* → *B*) needed for the MFPT in Eq 1 is obtained by summing over all steady-state probability flow into *B*:

$$\begin{array}{c} \mathrm{Flux}\left(A\to B\right)=\sum_{i\notin B}\sum_{j\in B}p_{i}k_{ij}\;\;, \end{array}$$ (3)

where the *k*_*ij*_ and *p*_*i*_ values are obtained solely from trajectories originating in *A*[28].

To accommodate a steady state, the boundary conditions of the weighted ensemble simulation described above must be slightly adjusted to induce a steady-state flow of probability from the initial state to the target state. This is accomplished by removing a trajectory from the ensemble whenever it enters the target state, and re-starting a new trajectory from the initial state with the same probabilistic weight as the one just removed. After a sufficient amount of time the system will relax into a steady flow of probability from one state to another, with probabilities in each bin maintained at a steady value. After this “burn-in” period, the Hill relation can be employed to estimate the MFPT.

Although not used in this report, we note that since the transition rates between bins can often be estimated accurately even before the probability distribution has relaxed to a steady state, a Markov-like transition matrix can be constructed and solved to infer long-timescale properties of the system, including the mean first passage time [28]. This approach is more efficient than waiting for the system to relax into a steady state when the probability mass itself is slow to relax, so long as there are sufficient transitions between bins, and the degrees of freedom orthogonal to the bins are either well-sampled or unimportant.

#### WESTPA implementation of WE

Throughout this work, we use the WESTPA [37] implementation of the weighted ensemble algorithm, which is freely available and open source (github.com/WESTPA). This implementation is flexible and adaptable for use with any stochastic dynamics engine, and supports plugins for extended methods such as the steady-state approach noted above. Interfaces currently exist for use with Gromacs, NAMD, AMBER, BioNetGen, and the present work provides one for MCell [37].

In order to simplify the process of using weighted ensemble sampling techniques with systems biology models, we have constructed an automated service to convert MCell models into ready-to-go WESTPA simulations, available at weightedensemblizer.csb.pitt.edu).

#### Comparing the efficiency of weighted ensemble and brute-force

There are different ways to characterize the gain in efficiency from using weighted ensemble instead of brute-force sampling. We find that a useful approach to evaluating efficiency, which is independent of specific computational architecture, is to take the sum of the simulated dynamics time in the weighted ensemble approach, and compare those results to simulating the same amount of dynamics in brute-force simulations. For instance, the single weighted ensemble run for the toy diffusive binding model presented in the Results section spawned a total of 610,704 trajectory segments in its 1000 iterations; as such, it is equivalent to simulating 610,704/1000 = 610.704 brute-force trajectories. Thus, always rounding up to give brute-force the benefit, we compare the weighted ensemble results to the results of running 611 brute-force simulations. The statistical precision exhibited by each method can then be compared on the basis of equal time spent simulating dynamics. As mentioned above, the overhead imposed by weighted ensemble resampling is very small compared to the time spent simulating dynamics for most systems of interest, so for models of even moderate complexity, we find this to be a fair comparison of efficiency.

Because WE trajectories, and hence observables, exhibit correlation within a single simulation, it can be important to perform multiple, independent weighted ensemble runs to ensure uncorrelated estimates of observables. When comparing the performance of brute-force sampling to multiple independent weighted ensemble runs, for each WE run we construct a brute-force ensemble of equivalent cost to each independent WE run, as described above. We can then compare the results of the multiple brute-force ensembles to the multiple independent WE runs on equal footing.

### Kinetic Monte Carlo for spatial behavior of biochemically active species: MCell

All simulations in this report employ spatially resolved particle-based kinetic Monte Carlo dynamics, implemented in the MCell software package. MCell (Monte Carlo Cell) is an open source program (MCell.org) that uses spatially realistic 3D cellular models and specialized Monte Carlo algorithms to simulate the movements and reactions of molecules within and between cells, or what is referred to as “cellular microphysiology” [38]. MCell has been used to study a wide range of neuroscience questions such as neurotransmitter diffusion in the brain [39], the structure and function of synapses in the central [40] and peripheral [29] nervous system, and the effect of drugs on nervous system function [41]. MCell has also been employed to investigate general cellular phenomena such as calcium signaling [42] and the role of diffusion in cellular transport [43].

MCell combines rigorously validated and highly optimized stochastic Monte Carlo algorithms, particle-based random walk diffusion of (point particle) molecules in space and on surfaces, and stochastic biochemical state transitions. MCell models can contain arbitrarily complex 3D mesh geometries representing the biological system under consideration. These geometries are typically derived from reconstructions of biological tissue (typically from electron microscopy data) [44], or created in silico based on average geometries [29], e.g. via CellBlender software (github.com/mcellteam/cellblender) [45]. MCell features a flexible model description language and has the ability to checkpoint simulation trajectories at arbitrary output intervals or times.

MCell is a *kinetic* Monte Carlo scheme, in the sense that the time evolution of the system is explicitly modeled. The Monte Carlo moves that the system makes are not arbitrary trial moves, but are rather chosen according to the reaction and diffusion rates of the molecules being simulated. A constant time-step is employed in these simulations, during which the likelihood of reaction and diffusion processes are computed and stochastically sampled; by using appropriate time-steps, the dynamics of the underlying processes are faithfully recapitulated (for further details, see [38, 46, 47]).

### Complex model construction: CellOrganizer and BioNetGen

The construction of large, complex spatial models is facilitated by a combination of software that specializes in separate aspects of this task.

One of the limiting factors in performing spatially realistic cell simulations is the difficulty of obtaining cell geometries. This limitation can be addressed by learning generative models of cell organization directly from microscope images; these can be used to synthesize an unlimited number of realistic geometries. For instance, in the complex model in a realistic cellular geometry studied below, biochemical reaction networks, with corresponding compartments for organelles, are constructed using BioNetGen software [48, 49], combined with cell geometry models generated by CellOrganizer software [50–58] using CellBlender [45] to create the MCell spatial simulations [59]. More information about this process of generating cellular instances with realistic cellular and subcellular organizations/morphologies is given below. The WESTPA software in turn manages ensembles of the MCell simulations, for either weighted ensemble or brute-force sampling.

CellOrganizer (CellOrganizer.org) is an open source tool for learning conditional generative models of cellular organization from images [50–58]. From these models, new cellular geometries can be generated from different parts of the “shape space” of the system. Currently CellOrganizer supports models for cell shape, nuclear shape, vesicle frequency, location and size, and microtubule length, number and distribution. Important for this work is CellOrganizer’s ability to produce realistic geometric instances of cells and subcellular components for use in modeling using the experimental spatial extension of the Systems Biology Markup Language (SBML) [60].

**Fig 3:**
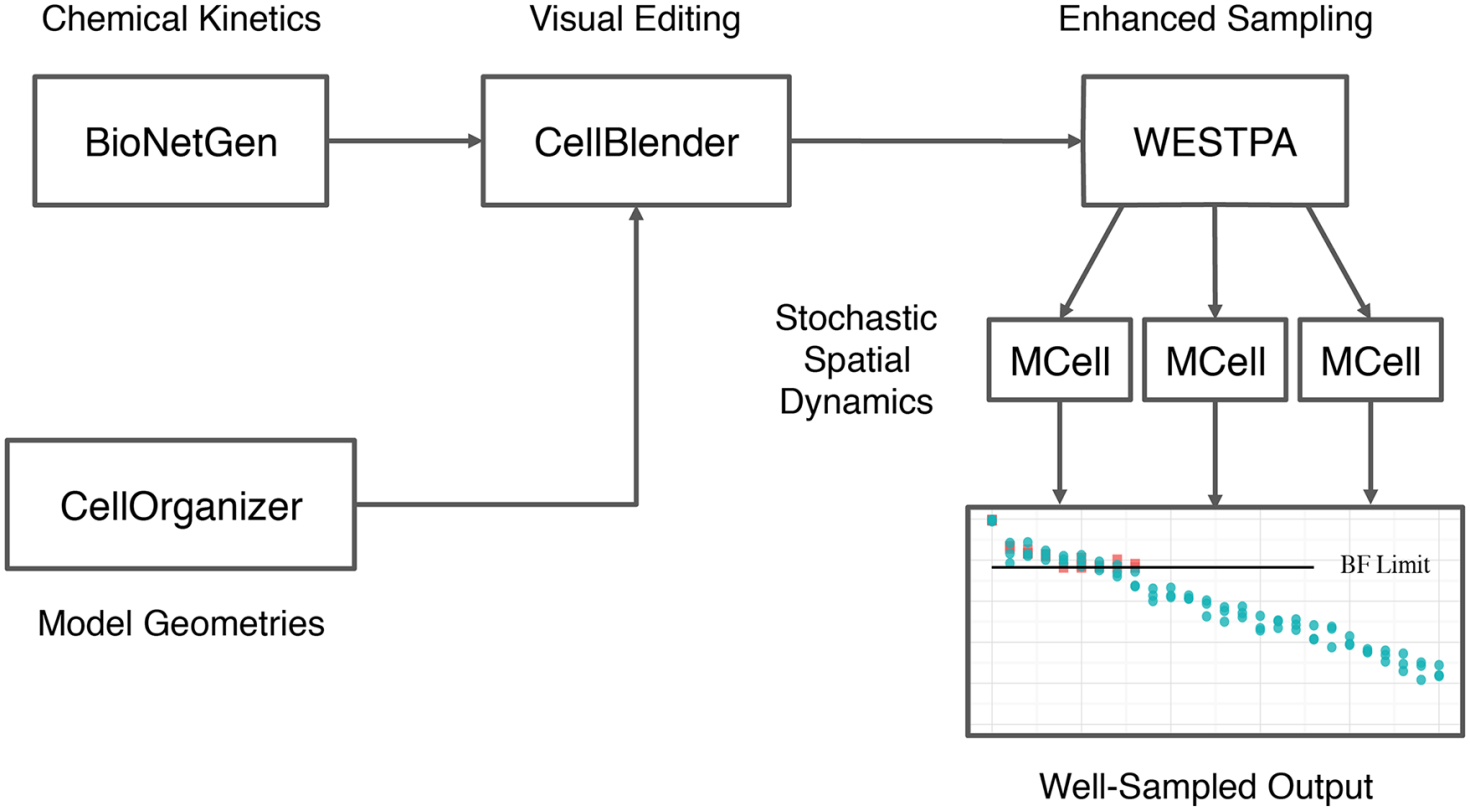
Software pipeline for realistic cell geometry simulations. Geometries are learned from images by CellOrganizer. Chemical reaction networks are generated from rule-based models in BioNetGen. Geometries and reaction networks are imported to MCell via the CellBlender visual editor. The spatial stochastic model is then simulated in MCell, with WESTPA managing a weighted ensemble of MCell trajectories.

Biochemical reaction networks in our model of signaling in a realistic cellular geometry are built with the BioNetGen software package (BioNetGen.org), which is a framework for specifying and simulating rule-based models of biochemical kinetics [48]. The rule-based approach allows combinatorially large chemical reaction networks to be compactly described using a small set of rules that define the underlying molecular interactions [49]. Indirect simulation of rule-based models requires automated generation of the reaction network implied by the rule set. The generated reaction network can then be simulated using a variety of approaches including ordinary differential equations and stochastic simulation. BioNetGen has previously been used to model a wide range of processes including signal transduction, metabolic pathways, and genetic regulatory networks [49]. BioNetGen enables the cellular topology to be defined via compartments [61], but it does not provide for the specification of more detailed geometric information about these compartments or molecule locations. An automated process converts these rules to an exhaustive network of chemical reactions representing the chemical kinetics of the system (see Fig 3).

The reaction network from BioNetGen is fed into CellOrganizer to obtain an appropriate cellular geometry, and the network and geometry are combined using the CellBlender package. In CellBlender, the reactions and geometry are merged, and exported to MCell. The system is then simulated as usual in MCell, either using weighted ensemble to manage the trajectories, or via brute-force.

## Models

We investigate three spatial models of cellular function: (1) a toy model of diffusive binding, (2) an idealized model of cellular signaling, and (3) a realistic model of a neuromuscular junction. All three particle-based kinetic Monte Carlo models are simulated in MCell (version 3.2.1), and are available in the supporting information.

### Toy diffusive binding model

**Fig 4:**
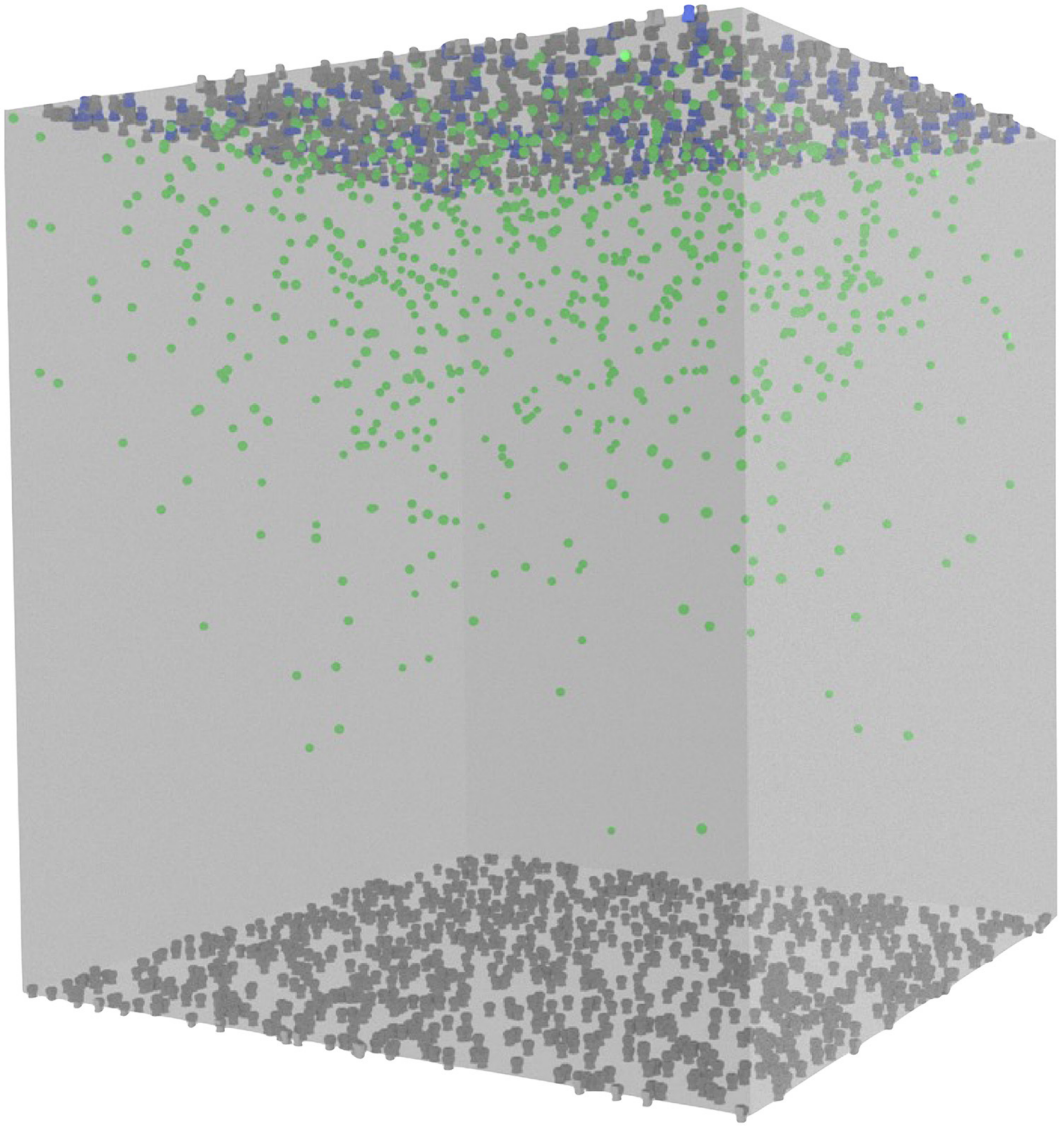
Toy model geometry. This toy system has receptors at the top and bottom of a cubical “cell”. The receptors at the top are initially bound by ligands, that are free to unbind and diffuse around the cell, and bind to receptors at the bottom.

A highly simplified model of diffusive binding was constructed as an initial test of the utility of weighted ensemble sampling in a spatial system. The model geometry is depicted in Fig 4.

In this toy model of diffusive binding, we define a cubical volume, of side length 2 microns, on the top of which 1000 ligands are initially bound to 1000 receptors at time *t* = 0. The volume also contains 1000 receptors at the bottom of the cube that are initially unbound. The ligands are then free to unbind (with a constant of 10^3^/sec), diffuse around the volume (with a diffusion constant of 10^−6^cm^2^/sec), and re-bind to receptors at the top, or to receptors at the bottom (with a constant of 10^8^/M/sec). We examine the probability density for the number of receptors at the bottom of the volume bound by ligands after simulating 10 milliseconds of dynamics.

#### WE simulation parameters

The toy model has an internal time step of 10 microseconds, and we perform weighted ensemble resampling at an interval that exactly coincides with the internal time step, or every 10 microseconds. We simulate the model for 10 milliseconds, or 1000 weighted ensemble iterations. The progress coordinate we use is the number of receptors bound at the bottom of the cube, with bins on this coordinate at integers on [0, 1000], and we simulate 16 trajectory segments in each bin.

### Complex model in realistic cellular geometry

There is significant interest in the variation of cellular morphology and its association with cell fate/function [59, 62–66], and here we employ a model that is a prototype for computationally investigating the effect of a specific geometry upon biological function. The system models protein production in response to an extracellular signal and highlights interesting aspects of signal transduction through different subcellular components, such as transport across membranes and feedback between molecules in different subcellular locations [59]. The model contains on the order of 10^5^ reactive molecules, situated in a realistic cellular geometry. Because creating robust, high-quality complex models of cells is itself a challenging endeavor, we employ the model generation pipeline through BioNetGen and CellOrganizer described in the Methods section and Sullivan et al. [59].

**Fig 5:**
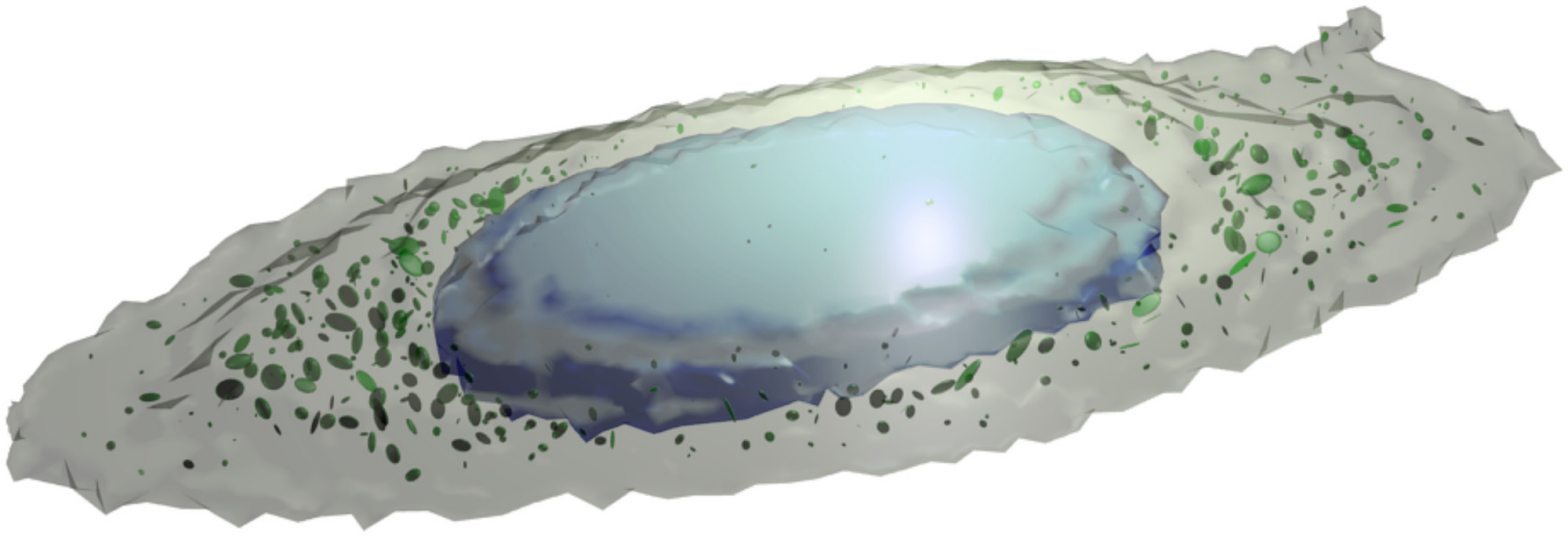
Cellular geometry. Realistic cell geometry generated from microscopy images by CellOrganizer. The geometry explicitly models the compartmentalization of the cell, by forcing molecules to diffuse through membranes to transition from, for example, the cytoplasm (grey) to the nucleus (blue). Also modeled are endosomes (green), and the extracellular environment (transparent).

We use the geometry shown in Fig 5, which is derived from three-dimensional images of HeLa cells using CellOrganizer. This geometry contains topologically distinct partitions: the extracellular region, the cytoplasm, the nucleus, and approximately 500 endosomes. The geometry also includes the membranes that partition these compartments, through which molecules must be transported when appropriate. Further details are included in the Supporting Information.

**Fig 6:**
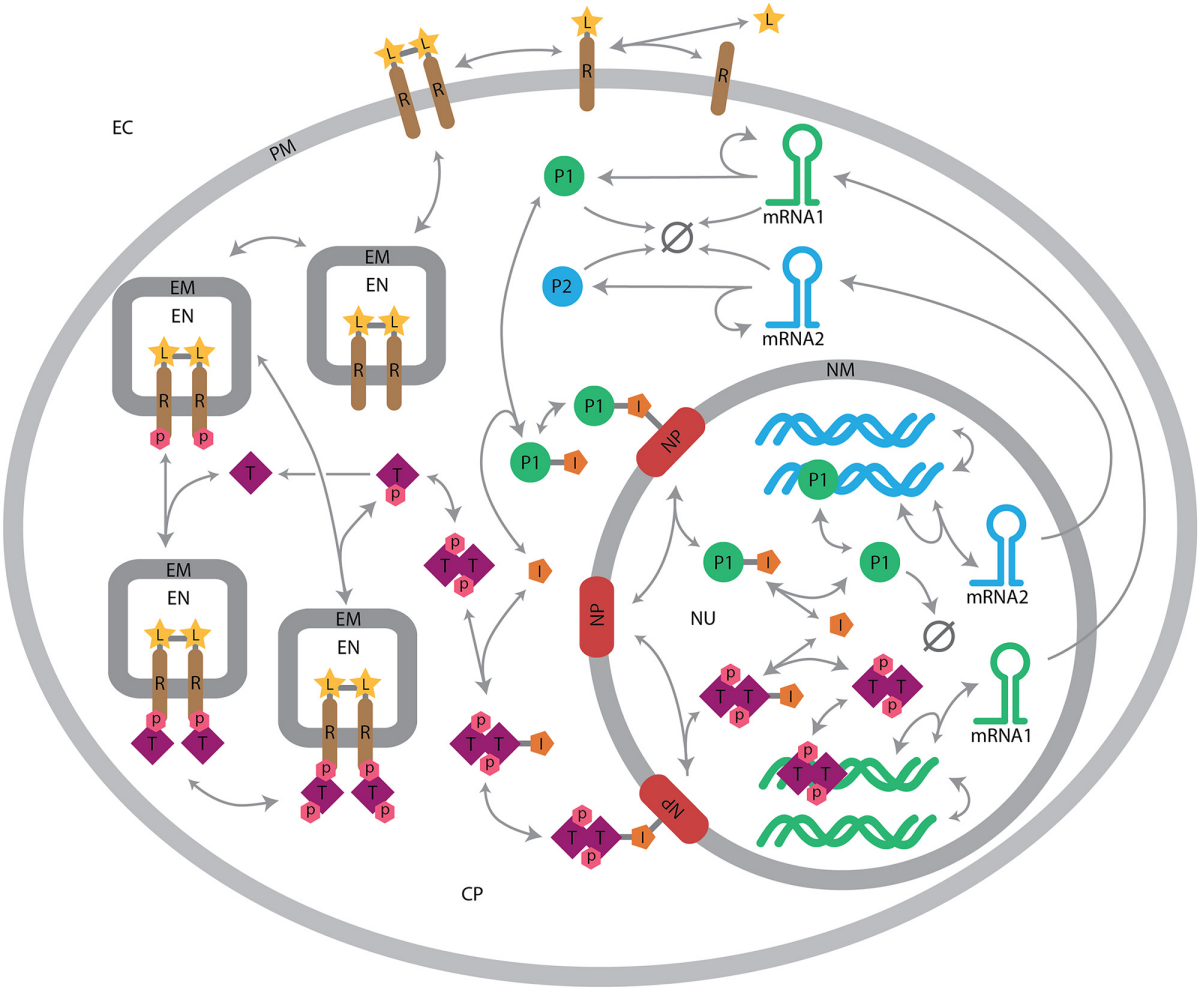
Signal transduction network. This rule-based model is translated into a system of chemical kinetics reactions by BioNetGen, and then simulated in a spatially realistic geometry by MCell. Figure adapted from [61]. Rate constants and further details are given in the SI.

We use the reaction schema illustrated in Fig 6 to describe the reaction kinetics of the model. The BioNetGen rules for this model are included in the Supporting Information, and they produce a network of 354 chemical reactions between 78 species [61]. Briefly, the signaling network functions as follows. The system is initialized in a state of unbound receptors, and free extracellular ligands. The extracellular ligand binds to receptors on the cell membrane, facilitating receptor dimerization, which can be internalized to the endosomes. In the endosomes, receptor dimers can become phosphorylated and recruit a transcription factor, which upon phosphorylation can also dimerize and migrate to the nucleus. In the nucleus, the transcription factor initiates the transcription of mRNA1, which, when it migrates to the cytoplasm, produces protein P1. P1 can then migrate to the nucleus and act as a transcription factor for mRNA2, which, when it migrates to the cytoplasm, produces the final species in the cascade, protein P2. Although this reaction network is idealized, it embodies key aspects of the complexity expected in real signaling processes.

#### WE simulation parameters

The weighted ensemble simulation of the spatial signaling model has an internal time step 100 microseconds, and we perform weighted ensemble resampling once every second, i.e. every 10^4^ internal time steps. We simulate the model for 500 seconds, or 5 million internal MCell time steps, i.e. 500 weighted ensemble iterations. We use a single progress coordinate for this system, the total number of P2 molecules in the system. The bins on this coordinate are integers on [0, 25] and one bin from 25 to infinity. We simulate 48 trajectories in the bin containing 0, and 16 trajectory segments in each other bin. Note that many coordinates (e.g., P1, ligands, mRNA1 and mRNA2, etc) are not divided into bins, as is typical of WE simulations of complex systems.

### Neuromuscular junction

The third model we study represents a single active zone of a frog neuromuscular junction (NMJ). Synapses are of crucial physiological importance in neural function, yet their detailed molecular behavior, particularly the way in which calcium triggers synaptic vesicle fusion still lacks a complete, molecular level, characterization. This is mainly due to the lack of experimental approaches that can probe synapses at the required spatial and temporal resolution. Computational models can provide critical microscopic insight into how calcium binding triggers vesicle fusion and release [29].

**Fig 7:**
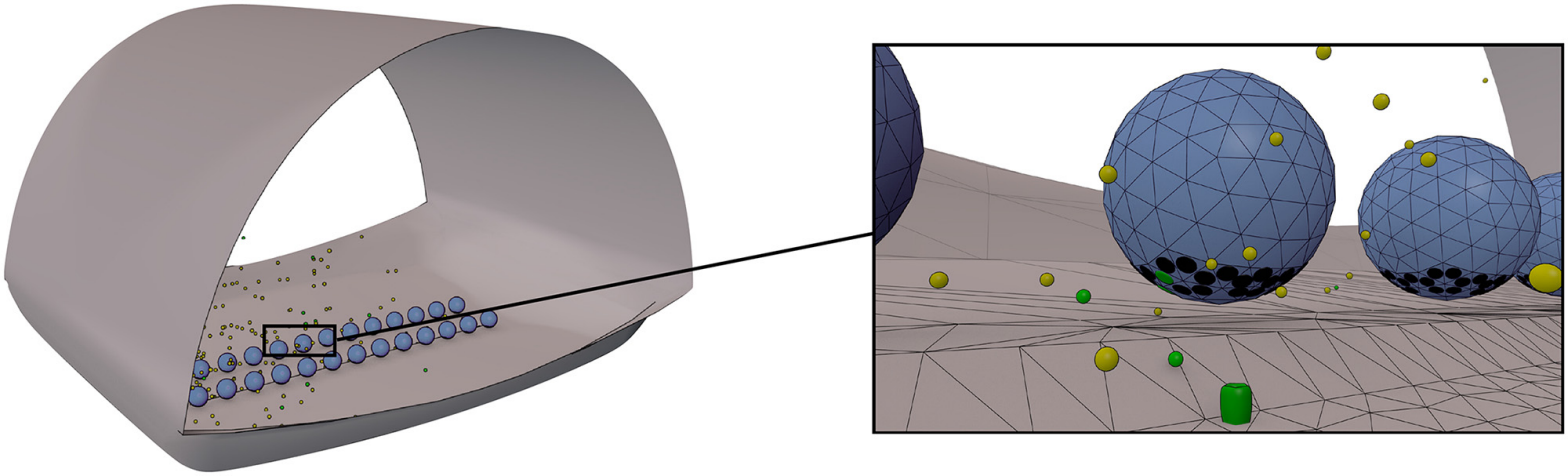
Schematic of the model of an active zone of a frog neuromuscular junction. On the left is an example snapshot from a simulation, and on the right is a zoomed-in view of the model. Calcium is released into the presynaptic space and is free to diffuse around the geometry and bind to the synaptic vesicles at the bottom of the active zone.

The geometry of the frog NMJ active zone model is detailed in Fig 7 and has been described previously [29]. The active zone model consists of a double row of 26 synaptic vesicles and two rows of 26 voltage gated calcium channels (VGCCs) in the space between vesicles (see Fig 7). Thus each synaptic vesicle is associated with a single VGCC.

The system is initialized from a state of no free calcium in the active zone. During a simulation, VGCCs open stochastically, driven by a time-dependent action potential waveform [29]. Once open, VGCCs conduct calcium ions into the presynaptic space. Calcium ions can then freely diffuse and either bind to ∼10^6^ static buffer molecules or one of eight calcium sensor proteins (synaptotagmin) on the synaptic vesicles. Since each synaptotagmin protein has five calcium binding sites, each synaptic vesicle contains a total of 40 calcium binding sites. A synaptotagmin protein is activated after binding at least two calcium ions, and vesicle fusion is triggered once three out of its eight synaptotagmin proteins have been activated. For each simulation we track the calcium binding events to synaptotagmin sites on synaptic vesicles and can thus determine the number of released vesicles and the time of release.

The NMJ model differs crucially from the two other systems studied here in that it possesses rate “constants” that vary in time. Specifically, the rates for the opening of and calcium conduction through VGCCs in the model are time dependent and are parameterized according to an experimentally measured action potential waveform. This time-dependent nature of vesicle release in synapses is critical for their physiological function [29]. Thus, the model, with its time-varying kinetics, cannot be treated using steady-state or equilibrium approaches and is only usefully simulated, even with a weighted ensemble, out of equilibrium and for a predetermined period of time.

#### WE simulation parameters

Weighted ensemble simulation of the NMJ model used an internal time step of 10 nanoseconds, and we performed weighted ensemble resampling at an interval of 6 microseconds for the low calcium conditions. In total, we simulate the model for 3 ms, i.e. 500 weighted ensemble iterations.

The progress-coordinate space for the NMJ system was two dimensional: one dimension was the total number of calcium ions bound to all synaptotagmin molecules on a vesicle, and the other was the number of activated synaptotagmin molecules on that vesicle. Since a vesicle fuses once three synaptotagmin molecules are active, the latter coordinate had integer bins from zero to three. For the coordinate tracking the number of bound calcium ions per synaptotagmin, the bins were integers on the interval [0, 20], and one bin from 20 to 40.

The NMJ progress coordinate was chosen to facilitate the observation of fusion events, in a manner that is somewhat complicated but also serves to illustrate the flexibility in the type of progress coordinates that WE accepts. Of the 26 vesicles in the simulation, the one that was closest to fusion was chosen at every WE iteration. That is, the vesicles were sorted in descending order by number of activated synaptotagmin proteins, and then by number of total calcium ions bound; the vesicle at the top of the list was chosen. This ranking was performed at every weighted ensemble resampling event, so in principle the vesicle in question could change during the simulation, but always in favor of progress towards a fusion event.

Due to the time dependent VGCC rate constants in the NMJ model, even weighted ensemble sampling can have difficulty efficiently filling up bins of state space. This is because some regions that are initially difficult to sample become easy to reach, and time spent populating intermediate bins is in some sense ill-spent—the model is still sampled, but the efficiency can be less than ideal if one attempts to always have all bins full of trajectories. To address this issue, instead of performing a single weighted ensemble run with a large number of trajectories, we perform many, less intensive weighted ensemble runs with fewer trajectories and average the results. Specifically, for the low calcium regimes of 0.5 and 0.3 mM in the Results Section for the NMJ model, we performed 100 independent weighted ensemble runs for each system. The 0.5 mM system maintained a target of 8 trajectory segments per bin, while the 0.3 mM system maintained a target of 16 trajectory segments per bin. As noted above, multiple independent weighted ensemble runs facilitate error estimation.

## Results

We sampled the three spatially resolved cell-scale models of varying complexity using the weighted ensemble approach. The results from all three models demonstrate the ability of WE to sample rare events in models of varying spatial and biochemical complexity. The application of WE sampling to the NMJ model generated novel data about vesicle release in regimes of calcium concentration too difficult to sample well with conventional methods.

### Toy diffusive binding model

**Fig 8:**
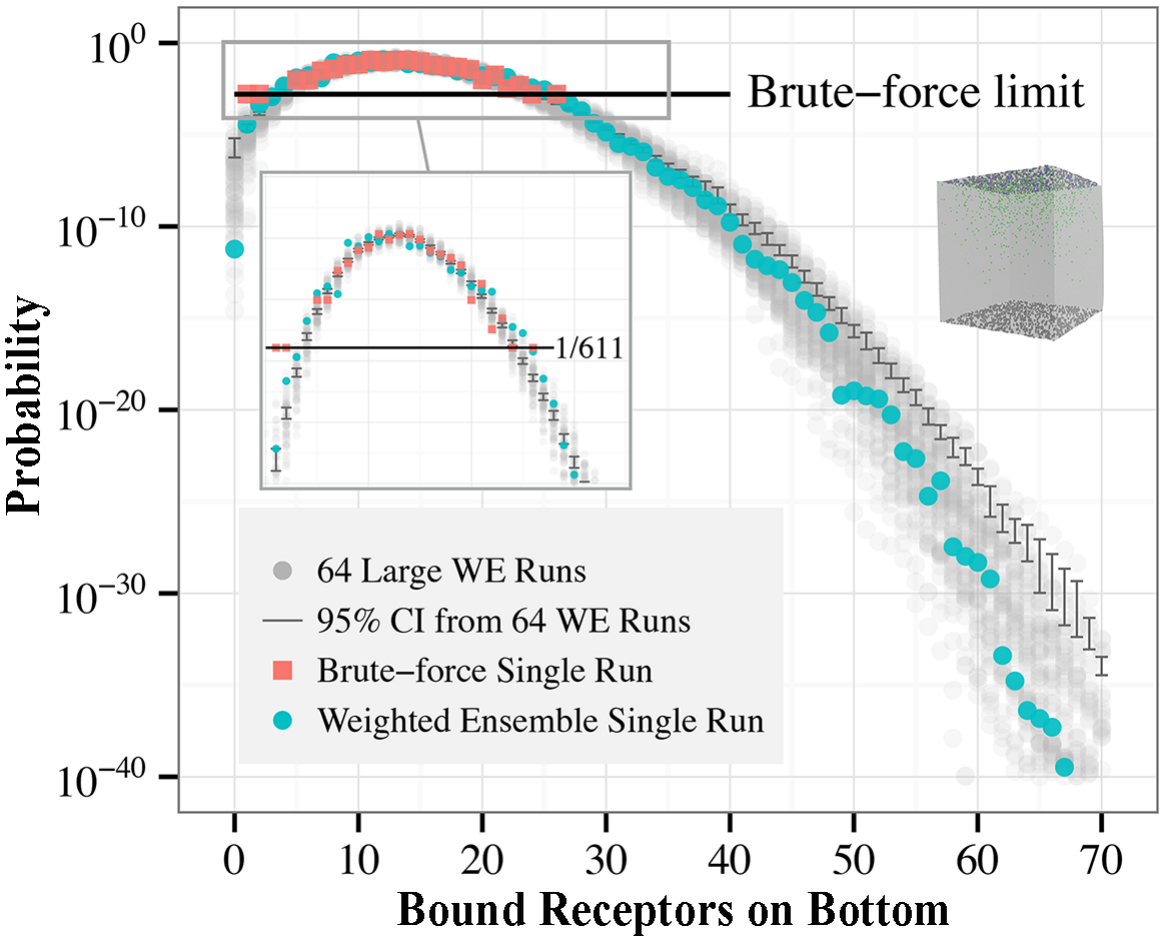
Sampling rare states of the toy binding model. Shown is the probability distribution of the number of bound receptors on the target side of the cell after 10 milliseconds of simulation. The weighted ensemble data (blue circles) is plotted with brute-force data (red squares) generated using equal computational effort, i.e. 611 brute-force runs. Brute-force sampling is confined to the peak of the distribution, whereas weighted ensemble sampling captures more of the full probability distribution. To compare both approaches to an authoritative value, an exhaustive set of 64 large weighted ensemble simulations was performed (grey circles), from which a bootstrapped 95% confidence interval for the mean probability distribution was calculated (dark grey bars).

Our studies of rare event sampling in spatial stochastic systems start with the toy model shown in Fig 4 and described in detail in the Models section. Briefly, we simulate diffusing ligands unbinding from the top of a cubical volume and binding to the bottom for a short amount of time. In this time-span, it is rare for a large number of the ligands to bind at the bottom of the volume. Indeed, when we simulate the system 611 times via brute-force, we see that in most cases only about 10–20 receptors are bound at the bottom after 10 milliseconds. We simulated 611 brute-force trajectories in order to make a fair comparison of weighted ensemble sampling to a brute-force approach; the single weighted ensemble simulation we performed required computational resources equivalent to 610.7 brute-force simulations. Looking more closely at Fig 8 (see inset), we see that it will be impossible to adequately characterize events rarer than 1/611 via the brute-force ensemble of simulations, since the rarest event one can see with brute-force is equal to the inverse of the number of trajectories. On the other hand, the weighted ensemble approach is able to sample the distribution over many orders of magnitude of probability with an equal amount of computational effort as the brute-force ensemble.

Since a toy model even this simple is too complex to solve exactly, we compare the data from both the single weighted ensemble simulation and the equivalent brute-force simulations to a more authoritative estimate of the probability distribution obtained by exhaustive (weighted ensemble) simulation. To obtain this reference value, we performed 64 independent weighted ensemble simulations with the same parameters as the single “test” weighted ensemble run (blue circles, Fig 8), except that each of the 64 runs had 32 trajectory segments per bin, rather than 16 for the test run (i.e. approximately 128 times the sampling power of the single run). From the 64 independent runs (gray circles, Fig 8), we then computed the 95% confidence interval for the mean probability distribution using 10,000 bootstrap samples at each progress coordinate, from 0 to 70. Even though the exhaustive weighted ensemble runs and the single test run use different weighted ensemble parameters (i.e trajectories per bin), this difference does not substantially affect the sampling quality of the ensembles. Note that the 95% confidence interval for the mean of the true distribution is significantly tighter than the variance of the distribution of weighted ensemble samples of that distribution; the fact that the single run falls outside this interval is typical of the stochastic noise inherent in a single WE sample.

As explained in the Methods section, weighted ensemble is able to sample more of the complete distribution by efficiently spreading out the sampling power of the ensemble of trajectories, allowing the characterization of rare-events by sacrificing some accuracy in the regime where brute-force samples well (see Fig 2). Examining Fig 8, we see that the brute-force distribution is smoother at the peak of the distribution—indicating less uncertainty—but only marginally so; the weighted ensemble estimate of the peak of the distribution is also reasonably smooth. By sacrificing unneeded resolution at the peak, WE is able to instead spread that sampling power more evenly throughout the state-space of the model, using it to sample the full probability distribution more comprehensively.

### Cross-compartmental signaling network in a realistic cell geometry

The model of cellular signal transduction shown in Figs 5 and 6 contains ∼10^5^, reactive molecules in a realistic geometry, and demonstrates the ability of the weighted ensemble sampling approach to scale to large, complex systems. We focus on characterizing the synthesis of protein P2. The production of P2, the last step in the cascade shown in Fig 6, is challenging to sample via brute-force. Nonetheless, it is a crucial quantity to calibrate if one is interested in the effects of spatial heterogeneity on the model, and we do so using weighted ensemble.

**Fig 9:**
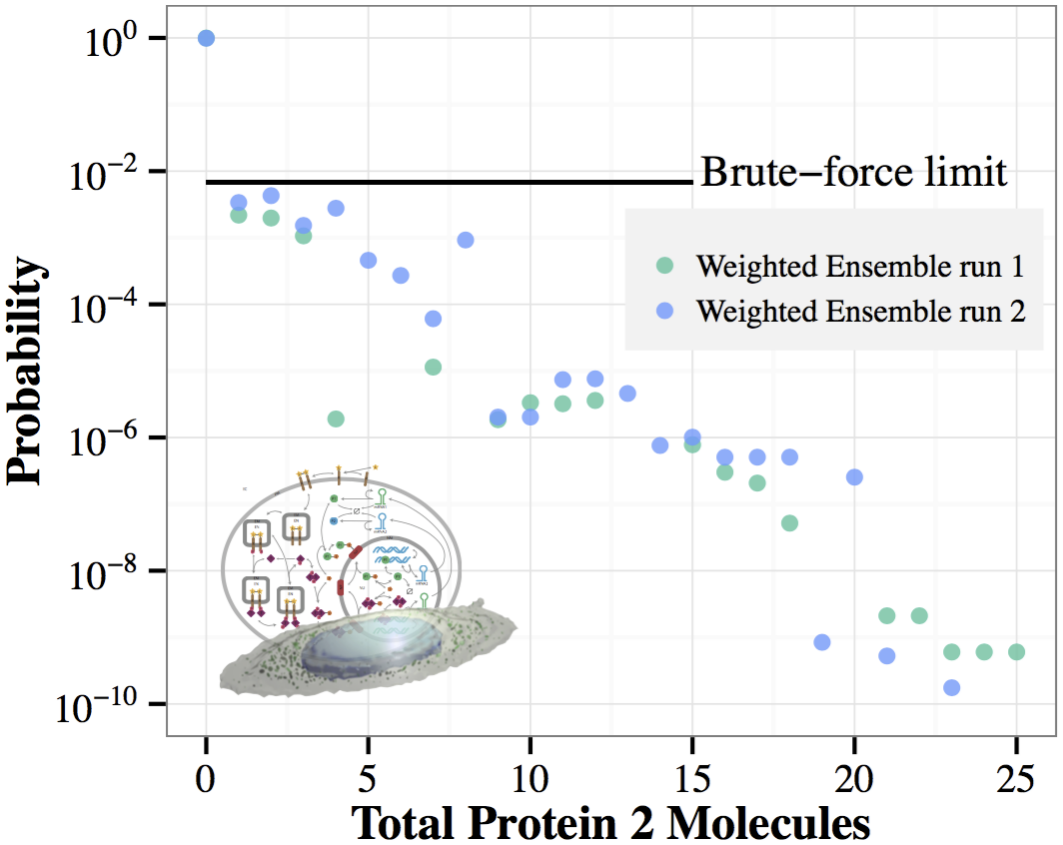
Accelerated sampling of high P2 levels. After 400 simulated seconds (∼1 week of wall time for one trajectory), we plot the histogram of the number of P2 molecules in the cell. The blue and green circles are the result of two independent weighted ensemble simulations. Note that some data points are missing, because not all weighted ensemble bins are necessarily populated by trajectory segments at all times.

To begin our exploration of the signaling model, we initially examine the production of the protein P2 after 400 seconds of simulation (see Fig 9). The weighted ensemble data was produced by two independent runs, and the two resulting independent histograms are shown together. The independent runs allow us to roughly characterize the uncertainty in the estimated probability distribution by simply inspecting the vertical spread in the results.

Detailed exploration of the tail of a probability distribution, as shown in Fig 9, can be interesting in its own right, for instance to detect multimodality, or otherwise explore the state-space for rare but important events. We are also interested in using the high resolution characterization of the tail of the P2 distribution as leverage with which to facilitate estimation of the *mean* time to the production of five P2 molecules. The target of five P2 molecules was chosen to represent a modest but non-trivial level of P2 production.

**Fig 10:**
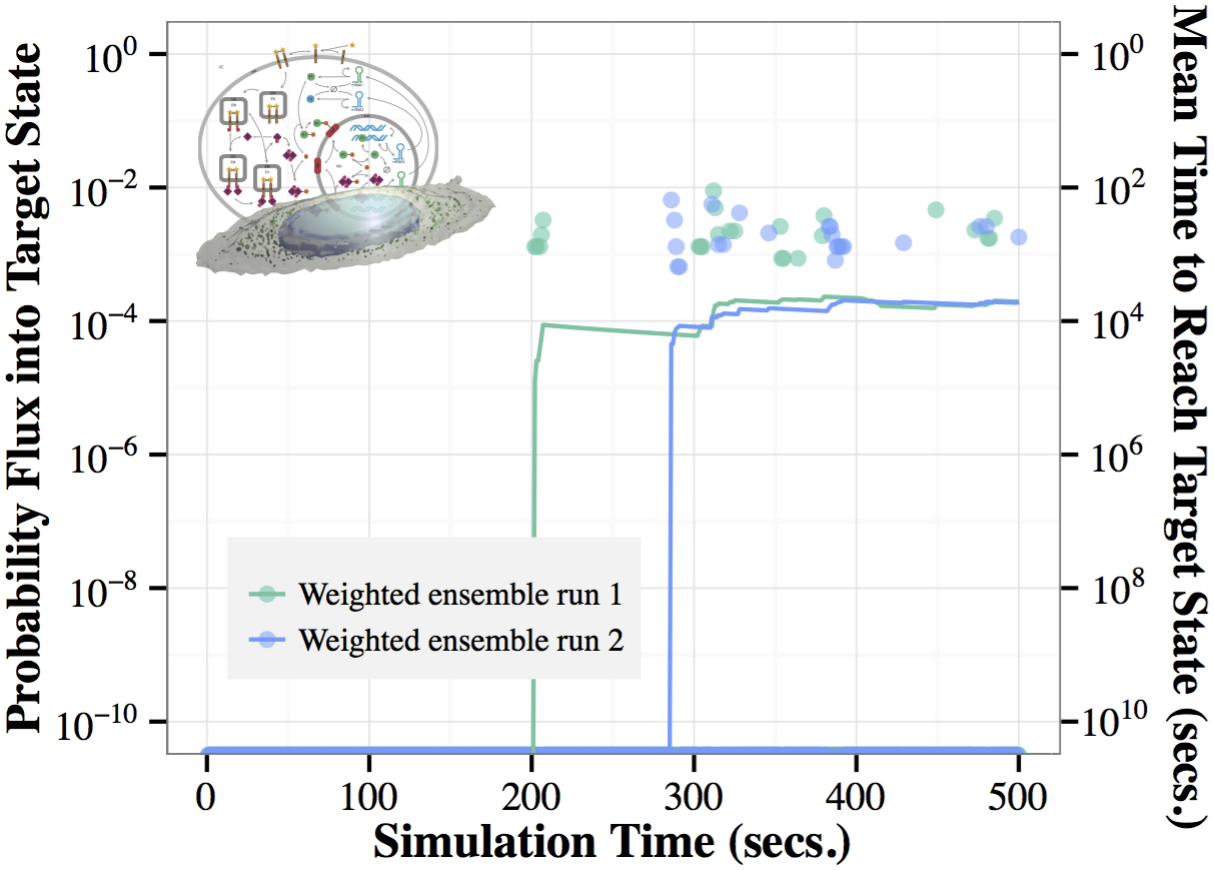
Steady-state estimate of time to produce five P2. The last event in the complex signaling network of Fig 6 is the production of P2. Using a steady-state approach, weighted ensemble is able to estimate the mean time to producing 5 molecules of P2 as approximately 5,000 seconds. The graph shows the probability flux arriving at the target state (left axis) in each iteration (points), and a running average of that flux computed from the most recent 50% of the data (lines). The mean time to reach the target state (right axis) is obtained via the Hill relation (Eq 1), as the inverse of the steady-state flux. Note that during most iterations, zero weight reaches the target state, as evidenced by the nearly continuous band of points at the bottom of the figure. The running average of the flux is dominated by these uneventful iterations, and hence is less than the nonzero instantaneous values.

To extract information about average P2 production time from short simulations, we work in a steady-state framework, as described in the Methods section. Using this methodology, we are able to infer the mean time to the creation of five P2 molecules, a relatively long timescale, from a weighted ensemble of short simulations. Shown in Fig 10 is probability flux arriving at the target state of five P2 molecules at each WE iteration, as well as a running average of those instantaneous measurements, made using the most recent half of the data up to that time. When the system reaches a steady-state, the inverse of the probability flux into the target state, shown for on the right vertical axis of Fig 10, is equal to the mean time to reach the target state. In Fig 10, we see that the estimated time to the production of five P2 molecules is on the order of 5,000 seconds. This estimate will converge, within stochastic noise, to the true MFPT of the system when the flow of probability induced by the recycling process has relaxed to a steady state; see the Methods section for details.

Although WE is extremely efficient at characterizing the P2 distribution (Fig 9), its performance for estimating the MFPT is not exceptional in this case. The two WE runs require 31,328 seconds (run 1) and 27,408 seconds (run 2) of aggregate simulation to reach the relatively steady estimation shown in Fig 10 at *t* = 400 seconds. By comparison, to obtain five to ten events for estimating the MFPT by brute-force sampling would require ∼25,000 to ∼50,000 seconds based on the estimated MFPT of ∼5,000 seconds. Note that such long runs would not be able to benefit from parallelization.

The efficiency of the steady-state approach to measuring the mean first passage time depends on the time to convergence, and the noise of the sampling, once converged. The noise of the sampling can be reduced by a more densely sampled weighted ensemble, but the time to convergence is more difficult to characterize. In the approach used here, the latter timescale depends on the waiting time to typical transition events (e.g. about 200 seconds in Fig 10), and the time it takes the system to relax to a steady-state. If these timescales (multiplied by the number of WE trajectories) are close to the timescale of the mean first passage time, then the estimate may not be particularly efficient. It will, however be less variable than a brute-force estimate of equivalent sampling power, and more convenient, in that it explores events of very different likelihood, and efficiently explores the state-space while estimating a key observable.

### Time dependent kinetics: Neuromuscular junction

Finally, we apply weighted ensemble sampling to a model of the active zone of a frog neuromuscular junction. This system, shown in Fig 7, and described in detail in the Models section, simulates the dynamics of vesicle fusion in the presynaptic terminal. The MCell model used in this study is identical to the one described previously [29]. Briefly, calcium molecules are released into the active zone, and are free to diffuse and bind to the calcium binding sites on the synaptic vesicles in response to an action potential. When enough calcium binds to a vesicle in the proper arrangement, the vesicle is considered to have fused.

Calibrating and validating the response of the model against experimental data is of crucial importance, but at low calcium concentrations, it becomes highly inefficient to perform brute-force simulation to gather good statistics. In the neuromuscular junction system, the probability of vesicle fusion depends on the external calcium concentration and falls off sharply as the calcium concentration is decreased.

**Fig 11:**
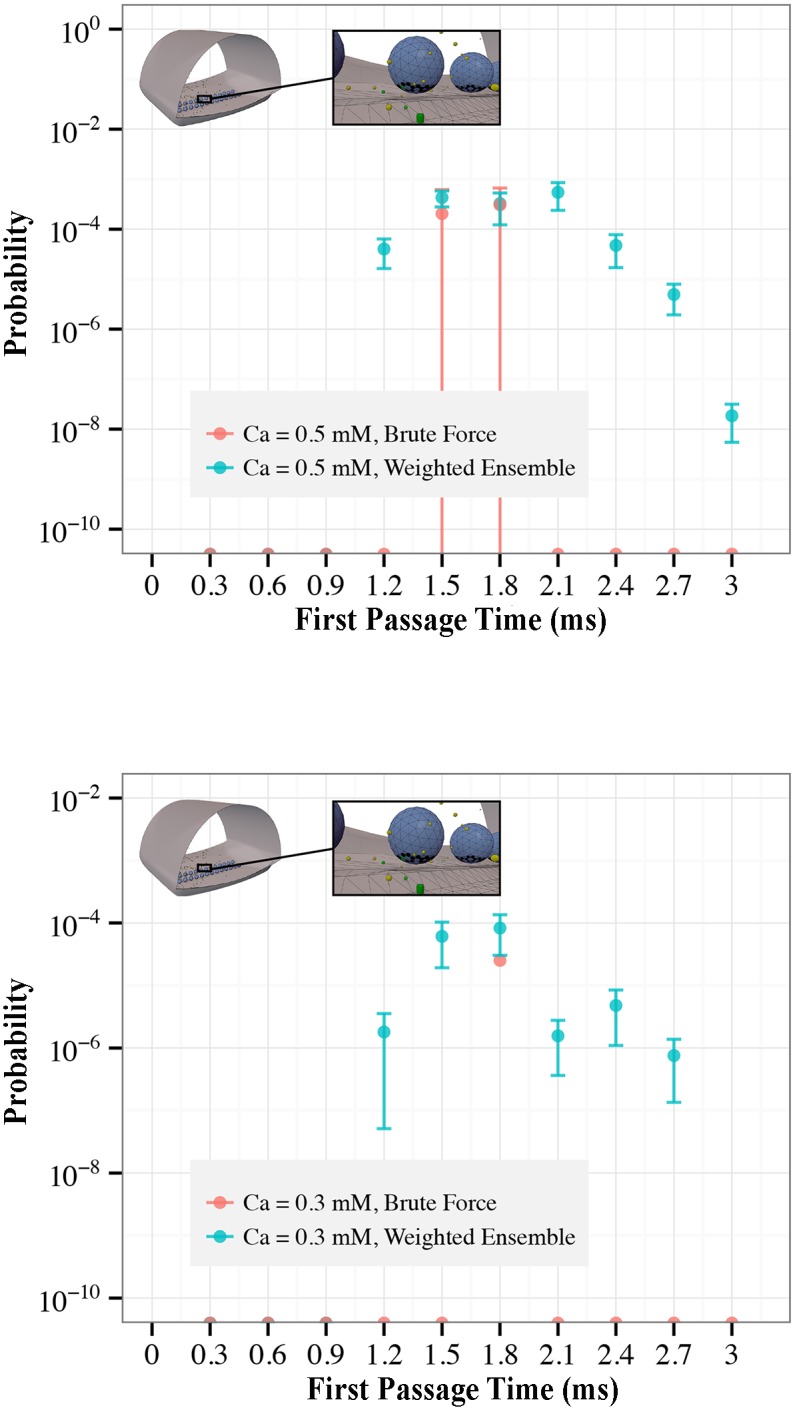
Enhanced sampling of the first fusion time distribution in the NMJ Model in low calcium conditions. Shown are measurements of the first fusion (i.e., first passage) time distribution for calcium concentrations of 0.5 mM (left), and 0.3 mM (right). In both plots, weighted ensemble estimate of the distribution of first fusion times for the NMJ model (blue), are compared to brute-force estimates (red) made using the same computational power as the weighted ensemble estimate. Points at the bottom of the plot indicate no fusion events in that time period. Single points with no error bars indicate only one sample, yielding no ability to estimate uncertainties. Brute-force sampling sees very few events, and gives a poor estimate of the shape of the distribution and yields poor confidence intervals (it is unable to exclude zero from any time point at one standard error). Weighted ensemble is able to capture the shape of the fusion time distribution, as well as providing good estimates in the uncertainty of the measured values.

Fig 11 shows the distribution of times to first fusion in the model, or first passage time (FPT) distribution to fusion, when the external calcium concentration is 0.5 mM and 0.3 mM. At each concentration, we plot the averaged results of 100 weighted ensemble runs, each of which was performed as specified in the Models section, as well as the averaged results of brute-force simulations, which in total required the same computational effort to simulate as the 100 weighted ensemble simulations (7545 brute-force simulations for the 0.3 mM system, 3513 for the 0.5 mM system). The difference with which the two approaches—weighted ensemble and brute-force —are able to capture the shape of the distribution, and the uncertainty in the estimation of it, is striking. We are unaware of any definitive methods of estimating error when the sample yield is extremely low, and hence have omitted error bars when only one or two samples were obtained.

At low calcium concentrations, the overwhelming majority of simulations do not result in a vesicle release, which is why brute-force sampling is so ineffective. Notice that the total area (i.e. the total probability of vesicle fusion) in the histogram for the 0.3 mM condition is only on the order of 10^−4^. One would have to perform on the order of 100/10^−4^ = 10^6^ simulations to start gathering meaningful statistics (100 samples) with which to compute the fusion time distribution. This amount of computing (∼20 years running in serial, if each simulation only takes a minute, or ∼20 weeks, running in parallel on a 48-core machine) is unfeasible to perform even once, let alone at all the different settings of model parameters of interest. Using weighted ensemble, however, it becomes practical to sample this model in the low-calcium regime, providing critical information for model validation and fitting purposes. The weighted ensemble sampling for the 0.3 mM condition shown in Fig 11 took time equivalent to 7545 brute-force simulations, and runs in matter of hours in parallel on 48 cores.

**Fig 12:**
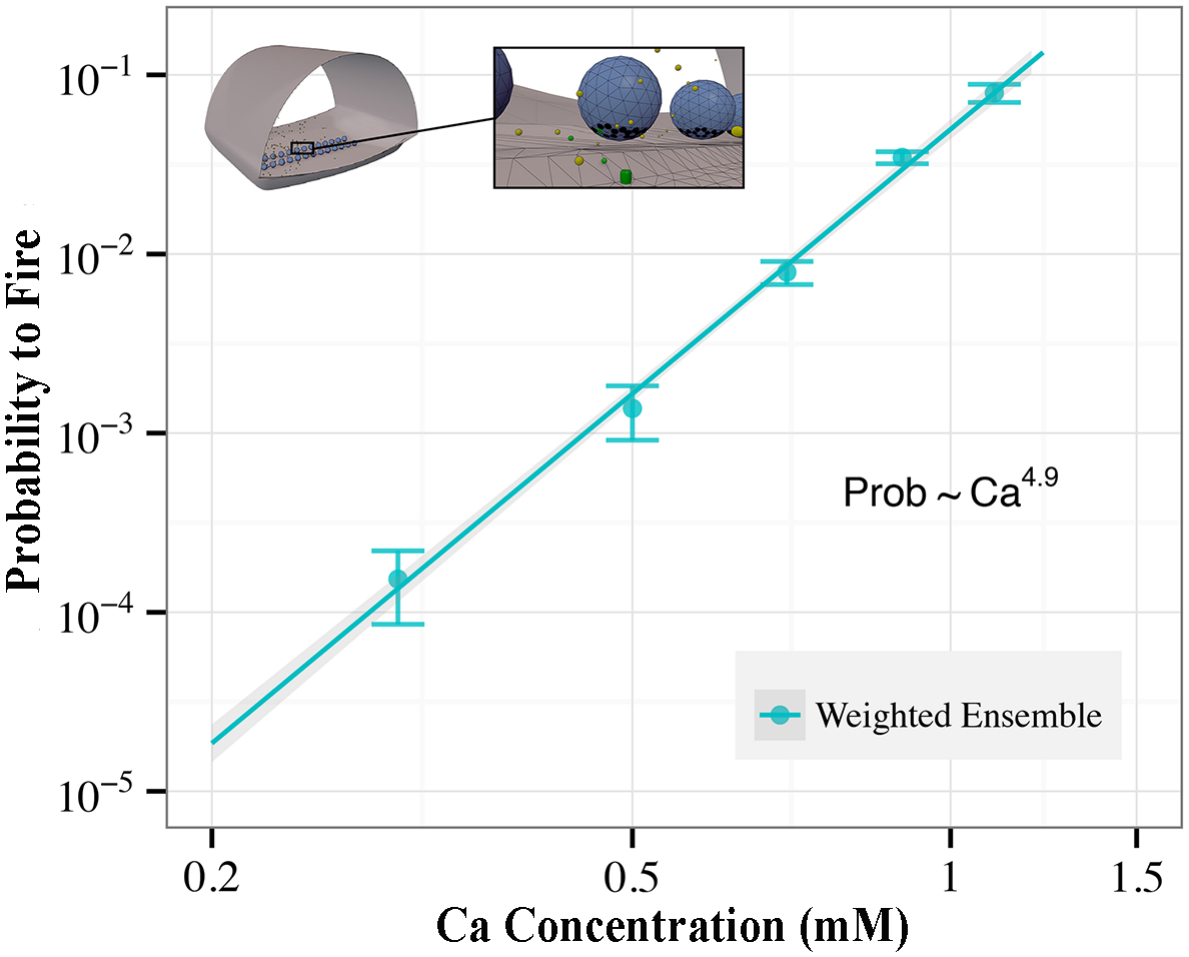
Verification of empirical fusion rate law extended to low calcium regime. The probability for the model to release a synaptic vesicle in the simulation window is plotted vs. the calcium concentration in the simulation. Weighted ensemble is able to efficiently estimate these probabilities in the low calcium regime (0.5 mM and below). WE data points and the power-law fit are shown with 1-*σ* confidence intervals.

Fig 12 summarizes NMJ results at five different experimentally relevant calcium concentrations. The data are a striking recapitulation of an experimentally demonstrated power-law dependence of probability to fuse as a function of calcium ion concentration [67]. Validating the model in low calcium regimes has been intractable with traditional sampling approaches. Using weighted ensemble, we are able to sample the model at all concentrations of interest.

## Discussion

Spatial models of stochastic reaction-diffusion processes have found widespread use as tools in understanding the mechanics of biological processes on the cellular level and beyond [68–72]. Unfortunately, the effective sampling of large, realistic models, and the extraction of well-sampled values of experimentally relevant quantities are often beyond the realm of computational feasibility. We use a weighted ensemble approach to overcome this impediment and demonstrate speedups of orders of magnitude in sampling some observables in complex models of cellular behavior with spatial dependence. Weighted ensemble is an ideal approach to employ in addressing the issue of difficult to sample stochastic systems, and because of its efficiency and ease of use, we anticipate many further applications.

### Strengths and weaknesses of WE

#### Strengths

Weighted ensemble is one of many enhanced sampling methods, and one of a smaller number that provides rigorous kinetics [17]. However, WE stands out from comparable approaches in its modularity, flexibility, and ease of use. Because weighted ensemble only performs resampling at fixed time intervals, and only when a trajectory transitions from one state-space bin to another, there is no need to catch trajectories in the act of crossing a state-space interface. This facilitates the implementation of weighted ensemble as a lightweight “wrapper code” around any number of simulation engines. A weighted ensemble approach also parallelizes trivially, as all trajectories are uncoupled while running, and are only compared intermittently during resampling. This efficiency in scaling has been demonstrated on simulations using over 2000 cores across many nodes on the Ranger supercomputer [37]. Additionally, a weighted ensemble of trajectories always follows the exact dynamics of the system; no biasing potentials, altered rate constants, change of measure, or other “hands on” tactics are necessary for efficient sampling.

A significant benefit of the WE approach is the ability to quickly find behaviors of a system that are very rare, or to detect the presence of multiple stable states of a system with high crossing barriers. As seen in our study of the cellular signaling model in a realistic geometry, efficient sampling via WE permits estimation of previously unknown long timescales using short simulations.

#### Weaknesses

All unbiased enhanced sampling methods that sample systems preferentially along a set of progress coordinates, including weighted ensemble, are useful only insofar as the state space has been divided along progress coordinates sufficient to characterize processes of interest. This limitation amounts to employing all of the slow, uncorrelated, degrees of freedom in the system as progress coordinates. Fortunately, an exhaustive use of all slow degrees of freedom is not required, as most will be correlated, and thus redundant descriptors of slow processes. Hence a “curse of dimensionality” does not cripple these approaches, and exists only to the extent that a system has important slow degrees of freedom which are uncorrelated with binned coordinates, but sufficient sampling is never guaranteed.

Sampling coordinates orthogonal to the progress coordinate will, by definition, not happen at an enhanced rate, which places a lower limit on the shortest useful simulations that can be performed. For instance, the toy model we investigate has only one effective degree of freedom, and displays an enormous amount of speed-up in sampling along this coordinate, because there are no slow orthogonal degrees of freedom being sampled on a slow, “native” timescale. On the other hand, the signaling model in the realistic cellular geometry shows less enhancement, a factor of about 10^5^ in sampling large amounts of P2, and less than that in estimating the mean time to production of five P2. This is because there are degrees of freedom in the system orthogonal to our progress coordinate that must relax to steady-state at an unenhanced rate, a process which occurs on a timescale uncoupled to weighted ensemble resampling.

A potential difficulty is that of correlation between trajectories. Since most WE trajectories share some history by construction, judging the degree to which related trajectories are independently sampling the space requires special care [24, 25]. Estimating observables within a single WE run requires careful consideration of time correlation. Alternatively, as in the present study, multiple independent WE runs directly provide unambiguous information on statistical uncertainty.

Weighted ensemble is not guaranteed to enhance sampling for all observables. In essence, WE will be most useful for the observables that occur with low probabilities on the time scales of interest. For less challenging quantities, such as the mean first passage time of Fig 10, or the high calcium concentrations of Fig 12, WE may primarily offer the advantage of simple parallelization.

### Summary and outlook

The multi-scale modeling problem posed by constructing accurate, physically realistic models of cellular level processes is considerable. We have demonstrated the utility of sampling spatially inhomogeneous stochastic simulations of cellular processes using a weighted ensemble (WE) approach. Although WE cannot estimate every quantity with high efficiency, estimates for some observables were obtained using orders of magnitude less overall computing than would have been required with conventional parallelization. We hope that these initial results will facilitate the study of more realistic and physically accurate spatial models of biological systems. As an ambitious example, integrating spatial models of stochastic processes with microscopy data of protein localization to predict phenotypic response to the perturbations of interactome networks is an attractive prospect for in silico drug development and personalized medicine. Currently, the bottlenecks in such a scheme are the lack of accurate models and the computational resources with which to simulate them. We hope that this work will contribute to the development of truly physiological computational models.

## Supporting Information

S1 Model Toy model. Files containing the model details and with which one can simulate the model in MCell.
(ZIP)

S2 Model Cellular model in realistic geometry. Files containing the model details and with which one can simulate the model in MCell.
(ZIP)

S3 Model Realistic cellular model reaction network. BioNetGen file containing the details of the reaction network used in the model.
(BNGL)

S4 Model Neuromuscular junction. Files containing the model details and with which one can simulate the model in MCell.
(ZIP)
